# Structural and Functional Neuroimaging of Polygenic Risk for Schizophrenia: A Recall-by-Genotype–Based Approach

**DOI:** 10.1093/schbul/sby037

**Published:** 2018-03-28

**Authors:** Thomas M Lancaster, Stavros L Dimitriadis, Katherine E Tansey, Gavin Perry, Niklas Ihssen, Derek K Jones, Krish D Singh, Peter Holmans, Andrew Pocklington, George Davey Smith, Stan Zammit, Jeremy Hall, Michael C O’Donovan, Michael J Owen, David E Linden

**Affiliations:** 1 Neuroscience and Mental Health Research Institute, Cardiff University, Cardiff, UK; 2 Cardiff University Brain Research Imaging Centre (CUBRIC), School of Psychology, Cardiff University, Cardiff, UK; 3 MRC Centre for Neuropsychiatric Genetics and Genomics, Institute of Psychological Medicine and Clinical Neurosciences, Cardiff School of Medicine, Cardiff University, Cardiff, UK; 4 MRC Integrative Epidemiology Unit (IEU), University of Bristol, Bristol, UK; 5 School of Psychology, Durham University, Durham, UK; 6 Centre for Academic Mental Health, Population Health Sciences, Bristol Medical School, University of Bristol, Bristol, UK

**Keywords:** schizophrenia, polygenic, recall-by-genotype, reward processing, imaging genetics

## Abstract

Risk profile scores (RPS) derived from genome-wide association studies (GWAS) explain a considerable amount of susceptibility for schizophrenia (SCZ). However, little is known about how common genetic risk factors for SCZ influence the structure and function of the human brain, largely due to the constraints of imaging sample sizes. In the current study, we use a novel recall-by-genotype (RbG) methodological approach, where we sample young adults from a population cohort (Avon Longitudinal Study of Parents and Children: N genotyped = 8365) based on their SCZ-RPS. We compared 197 healthy individuals at extremes of low (*N* = 99) or high (*N* = 98) SCZ-RPS with behavioral tests, and structural and functional magnetic resonance imaging (fMRI). We first provide methodological details that will inform the design of future RbG studies for common SCZ genetic risk. We further provide an between group analysis of the RbG individuals (low vs high SCZ-RPS) who underwent structural neuroimaging data (T1—weighted scans) and fMRI data during a reversal learning task. While we found little evidence for morphometric differences between the low and high SCZ-RPS groups, we observed an impact of SCZ-RPS on blood oxygen level-dependent (BOLD) signal during reward processing in the ventral striatum (*P*_FWE-VS-CORRECTED_ = .037), a previously investigated broader reward-related network (*P*_FWE-ROIS-CORRECTED_ = .008), and across the whole brain (*P*_FWE-WHOLE-BRAIN-CORRECTED_ = .013). We also describe the study strategy and discuss specific challenges of RbG for SCZ risk (such as SCZ-RPS related homoscedasticity). This study will help to elucidate the behavioral and imaging phenotypes that are associated with SCZ genetic risk.

## Introduction

Schizophrenia (SCZ) has a broad genetic architecture, characterized by thousands of common genetic variants (single nucleotide polymorphisms; SNPs)^1^ and rare pathogenic copy number variations (CNVs).^2^ These loci demonstrate biological convergence on central nervous system pathways such as voltage-gated calcium channel signaling, fragile X mental retardation protein (FMRP) gene targets, and excitatory/inhibitory synaptic neurotransmission.^3^ SCZ polygenicity also confers susceptibility to other psychiatric disorders, suggesting a shared, common etiology.^4^ While the cumulative (polygenic) effects of currently identified risk alleles explain approximately 7% of SCZ liability, these risk profile scores (RPS) do not yet offer predictive utility.^1^ However, SCZ-RPS are useful in identifying causal antecedents that predict disease risk such as reduced cognitive ability,^5^ increased substance use^6,7^ and higher incidence for specific SCZ symptom dimensions.^8^ SCZ loci also show genetic overlap with a number of polygenic, heritable traits, including personality, education and socioeconomic status.^9–12^ SCZ-RPS has also been combined with neuroimaging measures to identify disturbances in brain structure and function that reflect mechanisms of SCZ disease pathogenesis. These SCZ-RPS neuroimaging studies broadly suggest that the subcortical structural abnormalities observed in SCZ have little/no overlap with SCZ genetic etiology. However, behavioral and neural measures of cognition (eg, using functional magnetic resonance imaging [fMRI]) may reflect disease susceptibility.^13–17^ These imaging SCZ-RPS studies support theories that cognitive dysfunction is a risk factor for SCZ, while at least some of the alterations in subcortical brain volumes may be downstream effects of disease processes (reverse causation).^18,19^ Such genetic neuroimaging studies provide insight into the neurobiological mechanisms of SCZ, but are limited by sample size and heterogeneity.^20^

We first describe the recall-by-genotype (RbG) approach for neuroimaging SCZ-RPS. By assaying SCZ-RPS in a large, genotyped population, we are able to recruit a subset of individuals from the general population who have either extremely low or high SCZ-RPS, enriching the sample for a large amount of variation in SCZ-RPS, while minimizing problems with confounding and reverse causation that exist in samples of clinically ascertained individuals.^21^ As there is considerably increased SCZ risk (as indexed by odds ratio [OR]) in SCZ-RPS between the 1st and 10th decile,^1^ the current study offers considerably more power than an opportunistic sample (see Materials and Methods section).

In the current study, we assay neuroimaging phenotypes robustly linked to SCZ. There is reliable evidence that subcortical volume,^22^ cortical thickness/surface area are reduced in SCZ.^23^ There is also converging evidence that fMRI phenotypes, such as blood oxygen level-dependent (BOLD) signal relating to rewarding stimuli in the ventral striatum (VS) is altered in SCZ.^24^ However, it is largely unknown whether these alterations are linked to the common genetic risk for SCZ. Studies further suggest potential alterations in morphometric^25–27^ and VS-BOLD measures^28–31^ in relatives/offspring of patients with SCZ, suggesting putative familial effects. However, these studies cannot infer that common SCZ risk alleles explain these putative observations. Preliminary studies using SCZ-RPS suggest that the morphometric alterations observed in SCZ are largely not related to an individual’s burden of common SCZ risk alleles.^13,32,33^ In contrast, our preliminary work suggests common SCZ-RPS may explain some of the variance in the VS-BOLD response (as indexed by VS-BOLD).^14,34^ Together, these observations suggest that the SCZ-RPS is related to VS-BOLD but not morphometric measures such as subcortical volume. In the current study, we therefore aim to confirm the hypothesis that SCZ-RPS may influence fMRI phenotypes (such as VS-BOLD and across a wider network of previously investigated reward-related ROIs^14^), while morphometric measures (such as subcortical volumes) will remain largely unaffected.

## Materials and Methods

### ALSPAC Participants

The broader cohort sample from which we selected individuals consisted of young individuals recruited via the ALSPAC cohort. This broader cohort consisted of 14062 children born to women residing in the former Avon Health Authority area with an expected delivery date from April 1, 1991 to December 31, 1992 (http://www.bristol.ac.uk/alspac/; available at http://www.bristol.ac.uk/alspac/researchers/access/). Data were collected periodically from September 6, 1990, and collection is ongoing. Ethical approval for the study was obtained from the ALSPAC Law and Ethics Committee and the local research ethics committees (listed at http://www.bristol.ac.uk/alspac/researchers/research-ethics/).

### ALSPAC Participant Genotyping

All individuals recruited via the ALSPAC sample were genotyped using the Illumina HumanHap550 quad chip genotyping platforms by 23andme subcontracting the Wellcome Trust Sanger Institute, Cambridge, UK and the Laboratory Corporation of America, Burlington, NC. The raw genome-wide data were subjected to standard quality control methods. Briefly, individuals were excluded on the basis of gender mismatches; minimal or excessive heterozygosity; disproportionate levels of individual missingness (>3%) and insufficient sample replication (IBD < 0.8). Population stratification was assessed by multidimensional scaling analysis and compared with Hapmap II (release 22) European descent (CEU), Han Chinese, Japanese, and Yoruba reference populations; all individuals with non-European ancestry were removed. SNPs with a minor allele frequency of <1%, a call rate of <95% or evidence for violations of Hardy-Weinberg equilibrium (*P* < 5E-7) were removed. Cryptic relatedness was measured/excluded as proportion of identity by descent (IBD > 0.1). Related subjects that passed all other quality control thresholds were retained during subsequent phasing and imputation. Nine thousand one hundred fifteen subjects and 500527 SNPs passed these quality control filters. We combined 477482 SNP genotypes in common between the sample of mothers and sample of children. We removed SNPs with genotype missingness above 1% due to poor quality (11396 SNPs removed) and removed a further 321 subjects due to potential ID mismatches, resulting in a data set containing 465740 SNPs. We estimated haplotypes using ShapeIT (v2.r644) which utilizes relatedness during phasing. We obtained a phased version of the 1000 genomes reference panel (phase 1, version 3) from the Impute2 reference data repository (phased using ShapeIt v2.r644, haplotype release date Dec 2013). Imputation of the target data was performed using Impute V2.2.2 against the reference panel (all polymorphic SNPs excluding singletons), using all 2186 reference haplotypes (including non-Europeans). After quality control, a total of 8365 individuals were genotyped and underwent SCZ-RPS calculations.

### ALSPAC Participant SCZ-RPS Creation

Construction of the SCZ-RPS follows the methods described by the International Schizophrenia Consortium,^35^ using results from the Psychiatric Genomics Consortium (PGC) SCZ genome-wide association studies (GWAS).^1^ Polygenic scores were calculated for each ALSPAC individual using the “score” command in PLINK (version 1.07).^36^ Individual SCZ-RPS were created by summing the number of risk alleles present for each SNP (0, 1, or 2) weighted by the logarithm of each SNP’s OR for SCZ from the PGC summary statistics for each individual. Our SCZ-RPS–based RbG was solely based upon a RPS generated from SNPs with a GWAS training-set *P* ≤. 05 threshold, approximately 5% of all imputed SNPs. This threshold was specifically chosen as it captures the most SCZ liability (most variance explained) in the primary RPS analysis using training data/summary statistics derived from the largest SCZ GWAS of 34241 SCZ cases and 45604 controls.^1^

### SCZ-RPS Stratification and Cardiff Subsample

From the 8365 individuals who were considered for SCZ-RPS calculation, a total of 197 individuals (99 with low SCZ-RPS, 98 with high SCZ-RPS) participated in a battery of psychometric/neuroimaging paradigms, previously linked to the etiology of SCZ. A further 104 individuals declined our invitation (by written reply) to participate in the study (low SCZ-RPS [*n* = 40]; high SCZ-RPS [*n* = 64]), conforming to prior observations that SCZ-RPS is related to nonparticipation.^37^ Researchers were blind to which tail of the SCZ-RPS distribution each individual was selected from during the data collection and processing. All participants provided written informed consent. The SCZ-RPS groups were matched for gender (low SCZ-RPS: 52 female, 47 male; high SCZ-RPS: 52 female, 46 male).

### A Priori Power Analysis

Using the RbG approach, we estimated we had >80% power to detect an relatively small effect (*R*^2^ > .03), at a conservative alpha level (alpha > 0.001); see supplementary methods S1 and supplementary figure S1 for further details.

### Psychotic Experiences and Cognition

The semi-structured Psychosis-Like Symptom Interview was used to assess psychotic experiences (hallucinations, delusions, or experiences of thought interference) at 18 years of age.^38^ Individuals were deemed to have a psychotic experience if rated as having 1 or more suspected and/or definite psychotic experiences at 18 years of age (pliks18). Individuals were administered the short form Wechsler Intelligence Scale for Children (WISC-III) at 8 years of age.^39^ Scores for verbal, performance, and total IQ were taken forward for SCZ-RPS regression analysis.

### Statistical Analysis

Associations between SCZ-RPS groups and psychotic experiences were explored using Firth’s Bias-Reduced Logistic Regression via the logistf package in R.^40^ This approach computes confidence intervals computed by penalized profile likelihood to control for rare events. For WISC-III, Verbal, Performance, and Total IQ from the WISC were regressed against SCZ-RPS in a series of linear models. Gender was added into each model as a regressor in all cases.

### Structural Imaging Preprocessing and Analysis

Structural brain scans were acquired for each individual using a 3T GT HDx system at Cardiff University Brain Research Imaging Centre (CUBRIC), School of Psychology, Cardiff University. High-resolution 3-dimensional T1-weighted images were acquired using a 3-dimensional fast spoiled gradient echo sequence (FSPGR) with contiguous sagittal slices of 1 mm thickness (TR 7.9 s, TE 3.0 ms, TI 450 ms, flip angle 20°, FOV 256 mm × 256 mm × 176 mm to yield 1 mm isotropic voxel resolution images. Cortical and subcortical segmentations for each subject were estimated with well-validated segmentation software FreeSurfer version 6.0.^41^ In alignment with ENIGMA analysis strategies in SCZ and genomics,^13,22,23^ we explored (1) subcortical volume (mm^3^) (2) cortical (*a*) thickness (mm) and (*b*) surface area (mm^2^). Segmentations of 68 (34 left/right) cortical gray matter regions were created based on the Desikan–Killiany atlas and 7 subcortical regions (as well as the hemispheric total intracranial volume, average cortical thickness, and surface area). Segmented subcortical and cortical regions were visually inspected and statistically evaluated for outliers following standardized ENIGMA protocols (http://enigma.ini.usc.edu/protocols/imaging-protocols). For the statistical analysis, we averaged each segmentation/parcellation across hemispheres. Each ROI was regressed against SCZ-RPS group (low/high) with gender & ICV added as covariates. Age was not included as a covariate as all participants were born in the same year.

### Functional Imaging Acquisition and Preprocessing

Gradient echoplanar imaging data were acquired for each subject on the same 3T GT HDx system with an 8-channel receiver at CUBRIC (Cardiff University Brain Research Imaging Centre), School of Psychology, Cardiff University (parameters: 35 slices, slice thickness; 3 mm/1 mm gap, acquisition matrix; 64 × 64; FOV; 220 mm, TR 2000 ms, TE 35 ms, flip angle 90°, acceleration [ASSET] factor; 2). All functional images were first motion scrubbed, where TRs with a frame wise displacement >0.9 were removed, as previously recommended.^42^ Image processing and statistical analyses were conducted using statistical parametric mapping methods as implemented in FMRI Expert Analysis Tool (FEAT, Version 5.98, part of FMRIB’s Software Library, www.fmrib.ox.ac.uk/fsl). The following prestatistics processing was applied; motion correction using MCFLIRT^43^; slice-timing correction using Fourier-space time-series phase-shifting; nonbrain removal using BET (Brain Extraction Tool)^44^ spatial smoothing using a Gaussian kernel of FWHM 5 mm; grand-mean intensity normalization of the entire 4D data set by a single multiplicative factor; high-pass temporal filtering (Gaussian-weighted least-squares straight line fitting, with sigma = 50.0 s). Registration to high resolution structural (single subject general linear model [GLM]) and standard space (group-level GLM) images was carried out using FLIRT.^43^ Time-series analysis was carried out using FMRIB’s Improved Linear Model (FILM) with local autocorrelation correction.^45^ To further correct for any potential movement confounds, motion regressors were estimated via MCFLIRT and scrubbed TRs were added as covariates of no interest to each individual design matrix. After quality control procedures, 183 individuals out of the 197 (89 low SCZ-RPS and 94 high SCZ-RPS) where included in the reversal learning analysis.

### Functional Imaging Paradigm: Reversal Learning

Participants learned to choose 1 of 2 simultaneously presented colors (“blue” and “green”) by receiving monetary reward for correct choices and monetary punishment for wrong choices (eg, +1 pence [p] for “blue” and −1p for “green”). After 7–11 trials, reward/punishment contingencies were reversed so that the previously rewarded color was now punished and vice versa. Participants were instructed to maximize their earnings during the learning session, which consisted of 12 reversal episodes in total (108 choice trials). Within each reversal episode we included either 1 or 2 PE (probabilistic error) trials, in which “wrong”-feedback was given for correct choices, even though the reward contingencies had not changed. At the start of each choice trial, participants were presented with a response cue consisting of 2 white frames surrounding the colors and prompting the participants to press the left or right button on a response box to choose one color. Response feedback (choice outcome) was given subsequently using a centrally presented white “smiley” (correct choice) or red “frowny” (incorrect choice) face and an earnings counter changing incrementally by ±1p. In trials following reversal or PE events, ie, in those trials used for fMRI analysis, response cues and feedback stimuli were presented with a jittered duration (cue: 4–8 s, mean 5.5 s; feedback: 0.75 s followed by 3–7 s [mean 4.5 s] inter-trial-interval [ITI]: paradigm schematic in supplementary figure S2). To reduce scanning time, in all other (standard) trials we used fixed and shorter stimulus durations (cue: 2 s, feedback: 0.75 s). ITIs showed the 2 colors without response cue or feedback and were 0.5 s long after standard trials and between 4 and 8 s (mean 5.5 s) after PEs and reversals. BOLD response analysis focused on brain activation differences as a function choice behavior (switch > stay response) or choice outcomes (reward > punishment) in post-PE and postreversal trials. We selected those trials for analysis as they yielded a comparatively balanced number of rewards/and punishments (correct/vs incorrect choices) compared to standard trials (which were disproportionally more rewarded than punished. These regressors were modeled BOLD during decisional processes under high levels of uncertainty, ie, after participants had to choose a stay or switch strategy in response to an unexpected punishment in the previous (PE or reversal) trial and during rewarding or punishment based feedback. BOLD signal changes were regressed by task predictor functions (switch > stay and reward > punishment) convolved with a canonical hemodynamic response function. For the switch-stay contrast, predictor functions were synchronized with the onset of the response cue in post-PE/-reversal trials; having a duration of 4000 ms and including both predecisional and response processing. For the reward-punishment contrast and predictor time courses were locked to the onset of feedback stimuli in post-PE and postreversal trials, with a fixed duration of 3750 ms, which corresponded to the earliest possible start of the next choice trial. For each subject, statistical contrast images reward > punishment and switch > stay were obtained, which have previously shown good test–retest reliability.^46^ Group level analysis was carried out using FLAME (FMRIB’s Local Analysis of Mixed Effects).^47^ We explored the (1) group level contrasts (1-sample *t*-tests) and (2) SCZ-RPS group effects (2-sample *t*-tests) across (*a*) the whole brain, (*b*) within the ventral striatum (VS) region of interest, defined as the bilateral accumbens in the Harvard-Oxford Subcortical Structural Atlas, based on our previous observations between VS BOLD and SCZ-RPS,^14,34^ and (*c*) a preinvestigated reward network.^14^ For whole group analysis (1-sample *t*-tests) the family wise error (FWE) was controlled by estimating the minimum *Z* intensity using fsl’s “ptoz” function and contrast smoothness parameters, where *Z* > 4.2 controlled for the FWE across both choice decision and outcome. For between SCZ-RPS group comparisons, the family-wise error rate was controlled with nonparametric permutation testing (5000 permutations) and threshold free cluster enhancement (TFCE) which effectively controls for multiple comparisons, compared to cluster extent thresholding.^48^ The SCZ-RPS 2-sample *t*-tests for the switch > stay and reward > punishment contrast images, were adjusted for confounds (sex, relative, and mean frame wise displacement).

## Results

### Participant Stratification by SCZ-RPS

**Fig. 1.**
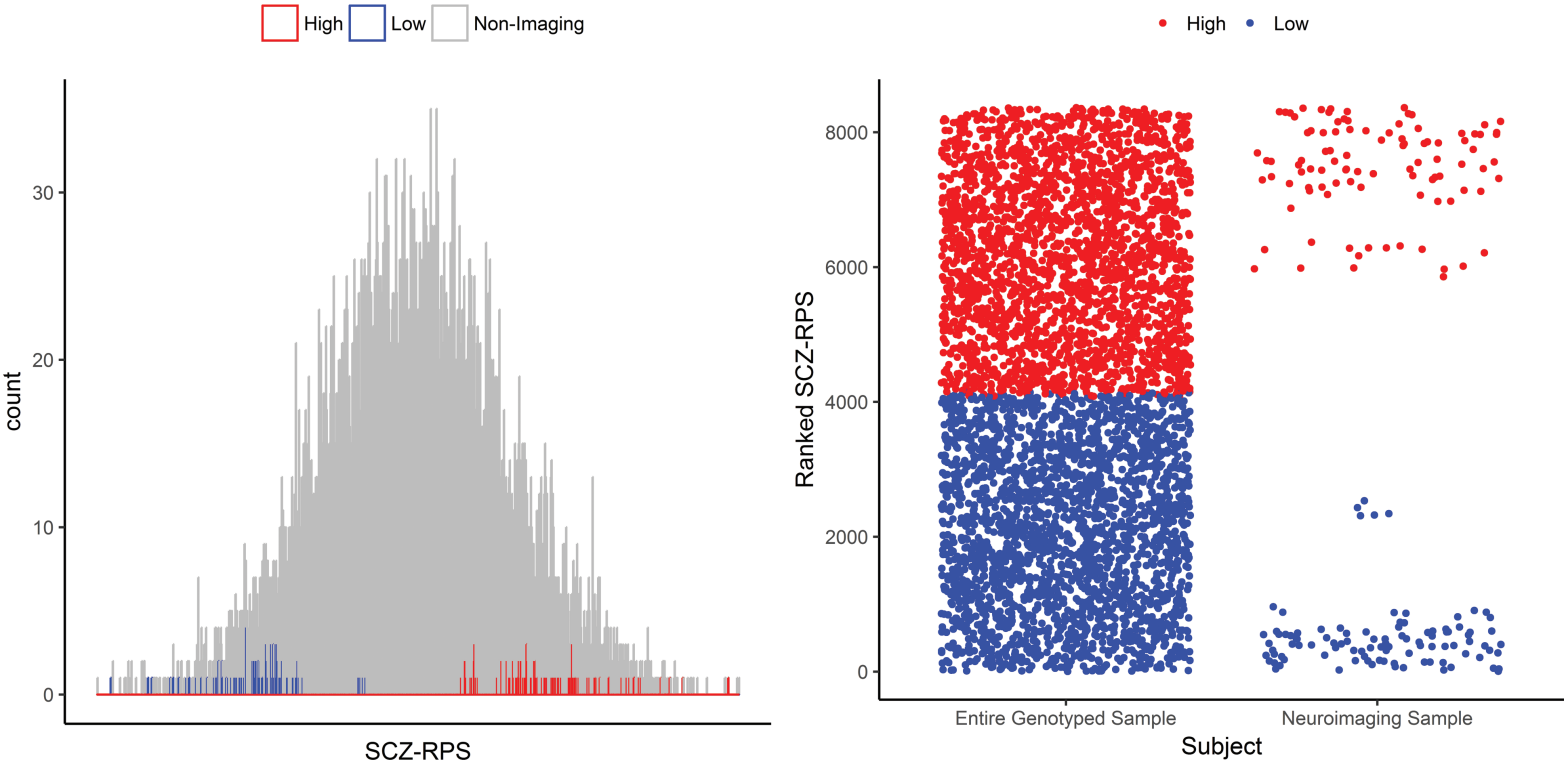
Characterization of the schizophrenia polygenic risk group in the neuroimaging sample (calculated by SCZ-RPS—left; defined by rank—right; *N* = 197; low = 99, high = 98) compared to the entire genotyped cohort (*N* = 8169, not including the neuroimaging sample).

We successfully phenotyped 197 individuals—99 (52 female, 47 male) individuals with low SCZ-RPS and 98 individuals (52 female, 46 male) with high SCZ-RPS from either tail of the SCZ-RPS distribution from a large, genotyped population (figure 1). There were also evidence of violation from homoscedasticity between the SCZ-RPS groups, where the cluster was more diffuse for the high SCZ-RPS compared to the low SCZ-RPS group (Levene’s test: *F*_1,195_ = 16.1, *P* < .001).

### Psychopathology and Cognition

For individuals where SCZ-RPS and psychotic experiences data were available (*n* = 172), we observed a nominal association between an increased incidence of psychotic experiences and high SCZ-RPS group allocation (psychotic experiences in low [*N* = 5; 5.75%] and high [*N* = 12; 17.65%] SCZ-RPS, *P* = .039). For individuals where SCZ-RPS and WISC-III measures were available (*n* = 183), we observed no association between SCZ-RPS and any IQ dimension (table 1).

**Table 1.** OR and β Coefficients (±95% Confidence Intervals) for Psychotic Experiences and WISC-III IQ Measures by SCZ-RPS Group (Higher OR/Coefficients Reflect an Association With the High SCZ-RPS Group)

Phenotype	Estimate	Lower 0.95%	Upper 0.95%	*P*
Psychotic experiences	1.100^a^	1.00660	1.20283	.039
WISC-III (verbal)	0.217^b^	−4.48233	4.91643	.927
WISC-III (performance)	1.944^b^	−2.83438	6.72296	.423
WISC-III (total)	1.606^b^	−2.77053	5.98341	.470

^a^Odds ratio (OR).

^b^β coefficients.

### Structural Neuroimaging

We observed no association between SCZ-RPS group and ICV, average thickness or total surface area (*P* > .1 in all cases). We observed nominal associations (*P*_UNCORRECTED_ < .05) between SCZ-RPS and cortical thickness in the superior parietal cortex and precuneus and between SCZ-RPS and surface area the caudal middle frontal gyrus (supplementary figures S3a and S3b), although these did not withstand correction for multiple comparisons. Regression analysis of the subcortical ROIs showed no association between the SCZ-RPS and subcortical volumes (supplementary figure S3c).

### Functional Neuroimaging

**Fig. 2.**
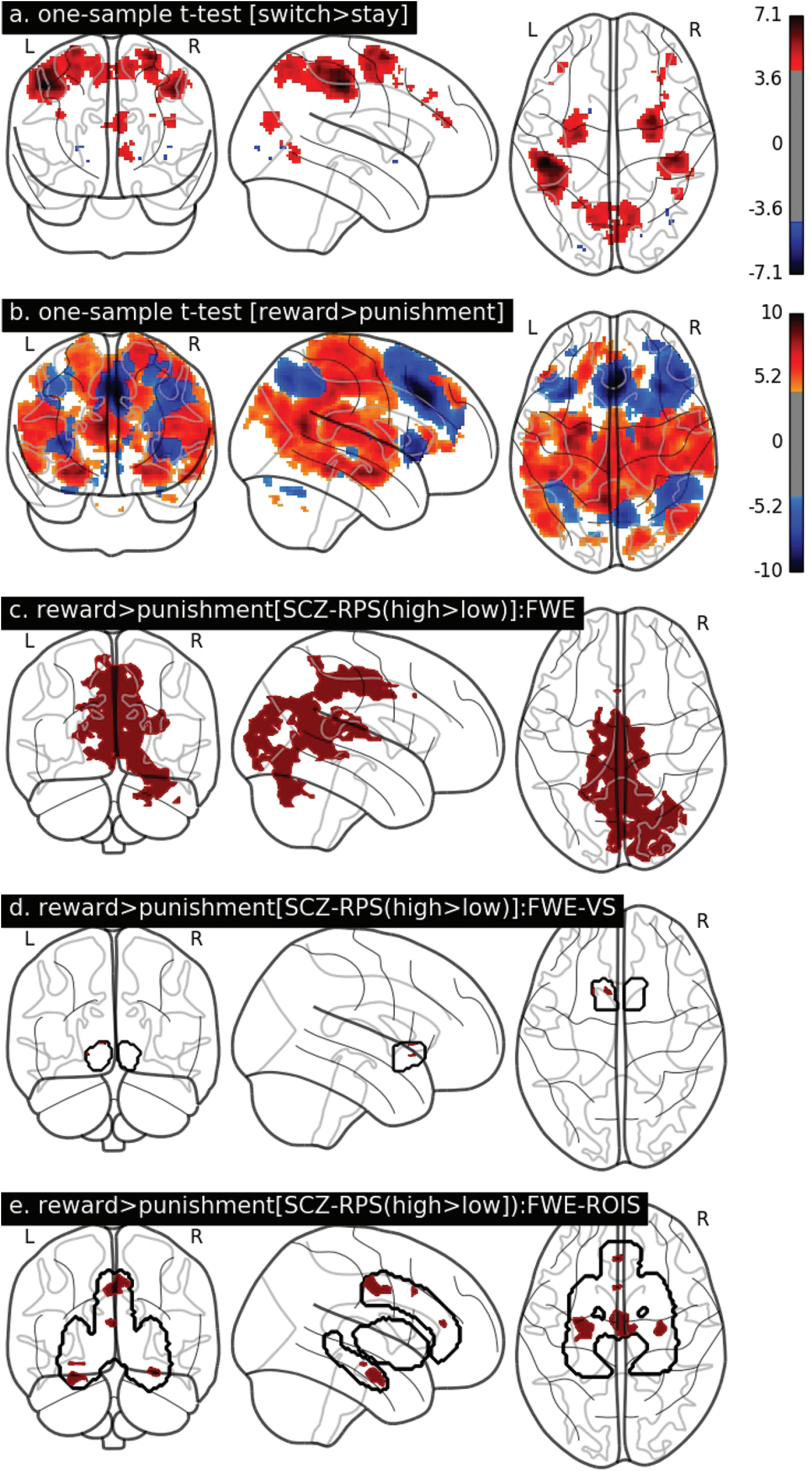
One-sample *t*-tests for (a) choice decision (switch > stay) and (b) choice outcome (reward > punishment). Both 1-sample *t*-tests are corrected for the family wise error across the whole brain (*Z* > 4.2). *Z*-map intensity is denoted by the colorbar for a and b. For the SCZ-RPS 2-sample *t*-tests, all significant voxels (non-white, within brain or region of interest boundaries) are corrected for the family wise error (*P*_FWE-CORRECTED_ < 0.05) across the (c) whole brain, (d) ventral striatum (VS), and (e) related-related region of interests (ROIs) adopted from Lancaster et al,^14^ all using threshold free cluster enhancement.

The combined group effects (1-sample *t*-tests) across all participants (for switch > stay and reward > punishment) produced similar *z*-maps as previously observed.^14,49^ Choice decisions (switch > stay) *Z*-maps were associated with BOLD signal increases in the bilateral precentral, postcentral, and superior parietal gyri. Choice outcomes (reward > punishment) *z*-maps was associated with a wide cortico-limbic network including superior frontal cortex, precentral gyrus, cingulate cortices, and hippocampal-amygdala complex (figures 2a and 2b, respectively). After correcting for FWE using TFCE correction (*P*_CORRECTED_ < .05), we found no effect of SCZ-RPS group in the choice decision contrast. However, we identified SCZ-RPS related group differences in the choice outcome contrast (reward > punishment) across the whole brain (*P*_CORRECTED_ = .013), VS-ROI (*P*_CORRECTED_ = .037) and reward-related ROIs (*P*_CORRECTED_ = .008), controlling for confounds (figures 2c–e, respectively), where the high SCZ-RPS showed higher BOLD than the low SCZ-RPS in both cases. The low and high SCZ-RPS groups were matched for performance (accuracy [% correct] and/reaction time) in the post-PE and postreversal trials where choice decision and outcome where modeled (supplementary table S1, and supplementary figure S4).

### Brain–Behavior Relationships

We then investigated whether the SCZ-RPS–related variation in BOLD (figures 2c–e) was related to the SCZ-RPS–related variation in psychotic experiences that we observed. In a series of linear regression models, we found no evidence for association between SCZ-RPS related BOLD in any of the clusters identified within the whole brain or ROI analysis (*P* > .1, in all cases). We further averaged performance across postprobabilistic error and postreversal trials and found no relationship BOLD in any SCZ-RPS related brain regions (*P* > .1, in all cases).

## Discussion

We first outline an RbG strategy for the deep phenotypic characterization of healthy, young individuals with either a low or high burden of common risk alleles for SCZ, as estimated via SCZ-RPS. There was more variation in SCZ-RPS in the high SCZ-RPS group, due to difficultly in participant recruitment in this SCZ-RPS group, consistent with the previous observation that higher SCZ-RPS is associated with a higher incidence of participation attrition/nonparticipation.^37^ Our finding of a nominal association between SCZ-RPS and psychotic experience are similar to a recent observation (supplementary eTable 3 in Jones et al^8^). It is currently unknown whether SCZ-RPS reflects a specific risk to develop psychotic symptoms or relates to broader psychopathological constructs such as common mental distress.^50,51^ Recent work aimed to uncover the relationship between SCZ-RPS and specific facets of SCZ psychopathology,^8^ however this work is ongoing.^52^ The lack of replication of the association between SCZ-RPS and IQ may have been observed as our study was not powered to find effects of the size previously reported.^5^

We also provide an analysis of putative structural brain differences (volume, thickness and surface area) between the SCZ-RPS groups. We found no effect of SCZ-RPS on whole brain measures (ICV, cortical thickness or total cortical surface area), which is likely to rule out any shared variance at the level (*R*^2^ > .03) for which our study was powered. While we observed no relationship between SCZ-RPS and brain morphometry (in concordance with other SCZ polygenic imaging studies^13,32,33,53^), several studies suggest that associations between SCZ-RPS and brain structure may be region specific^54^ or interact with other risk factors,^7,55,56^ which remain relatively unexplored. In our sample, there were nominally significant differences between groups for cortical thickness and surface area in parietal and frontal brain regions; however these observations should be confirmed in independent studies before we can assess their role in the etiology of genetic risk for SCZ.

Critically, we observed an impact on SCZ-RPS on BOLD during reversal learning, while performance remained intact. SCZ-RPS was related to increased BOLD in the (1) VS, (2) extended reward-related search space, and (3) across a broader cortical network—extending into posterior regions of the brain. While there are similarities between these observations (eg, increased VS-BOLD and SCZ-RPS during reward receipt^34^), we observed several differences between the current findings and our previous findings. Specifically, we observed altered BOLD signal during the processes of choice outcome (ie, during reward receipt), compared to our previous observation linking SCZ-RPS to BOLD during choice decision (ie, uncertainty of outcome). However, we suggest that these observations largely conform to our broader hypothesis that BOLD signal in the reward processing network is associated with SCZ-RPS.^14,34^ We expand upon our previous findings by demonstrating that the altered BOLD signal associated with increased SCZ-RPS extends across a wider network including the hippocampal, cingulate cortex, precuenus, and thalamus. Imaging studies of individuals with increased genetic risk for SCZ have also implicated these cortical/subcortical regions,^57,58^ which may reflect the recruitment of alternative/additional neural resources proposed for other SCZ-associated fMRI-based endophenotypes.^59^ In line with previous hypotheses,^18,19^ these observations suggest that the common genetic architecture of SCZ may manifest via alterations in the activity of cognitive-motivational brain networks (eg, supporting reversal learning), in the presence of a relatively intact cortical/subcortical morphometry. This hypothesis is also supported by studies showing common genetic overlap between SCZ and cognition,^5,11^ but not brain volumes.^13^ Although we find evidence supporting SCZ-RPS related alterations in VS-BOLD in healthy individuals, it remains unknown how these alterations predispose risk to SCZ. Studies suggest that SCZ-related alterations in VS-BOLD could relate several symptom dimensions including (1) myopic decision making (similar to the VS-BOLD alterations observed in ADHD)^60–62^; (2) positive symptoms (such as delusions/aberrant salience)^63–66^ or (3) negative/depressive symptoms (such as anhedonia/avolition).^24,67–69^

Our future objectives are to explore the SCZ-RPS group differences across a range of neurophysiological and connectivity measures. We anticipate that these analyses will further elucidate the brain systems that are linked to the common genetic architecture of SCZ. In a secondary analysis, we hope to further establish specific SCZ biological pathways (such as glutamate receptor complexes, voltage-gated calcium channels, FMRP binding proteins^3^) that may preferentially influence these putative associations. By identifying specific neural antecedents, we aim to provide novel biological insight into brain systems (and associated psychopathological symptoms) disrupted in SCZ.

In conclusion, we provide a framework by which to explore the impact of RPS on quantitative neural and behavioral traits. This approach offers the statistical power of a large genotyped population study, without the cost of extensive phenotypic characterization. This method could also be used in other systems-biology approaches such as the neuronal conversion and phenotypic characterization of low/high SCZ-RPS human fibroblasts, classify the efficacy of response in clinical trials and psychological intervention programs.

## Funding

This work was supported by grant MR/K004360/1 from the Medical Research Council (MRC) titled: “Behavioural and neurophysiological effects of schizophrenia risk genes: a multi-locus, pathway based approach” and by the MRC Centre for Neuropsychiatric Genetics and Genomics (G0800509) and the NIHR Bristol Biomedical Research Centre. The UK Medical Research Council and Wellcome (grant ref: 102215/2/13/2) and the University of Bristol provide core support for Avon Longitudinal Study of Parents and Children. GWAS data were generated by Sample Logistics and Genotyping Facilities at Wellcome Sanger Institute and LabCorp (Laboratory Corportation of America) using support from 23andMe.

## Supplementary Material

Supplementary Material
