# Supplementary material for: Structural and Functional Neuroimaging of Polygenic Risk for Schizophrenia: A Recall-by-Genotype–Based Approach
Source: Schizophr Bull. 2018 Mar 28;45(2):405–14. doi: 10.1093/schbul/sby037 (PMC6403064; doi:10.1093/schbul/sby037)
Supplement: Supplementary Material [file sby037_suppl_supplementary_material.doc]

**Supplementary Methods**

**sM1. *A priori power analysis***

Power was estimated by simulating two independent random standard normal variates X and Y, and constructing a variable Z=X+bY. Here, Y represents the SCZ-RPS and Z the quantitative phenotype being tested. The proportion of phenotype variance accounted for by the polygenic score was denoted as ‘b’ (square root of (R2 / (1+R2)). 197 samples were matched based on their actual SCZ-RPS rank, to reflect the selection procedure from the ALSPAC data, where 8,365 individuals were available in the ALSPAC sample. The correlation between Y and Z is then tested in these selected samples, and power defined as the proportion of simulated samples achieving the required alpha level. We also randomly selected 197 individuals from the sample to compare power of the RbG approach, compared to an opportunistic sample. For the targeted analysis of associations between polygenic scores and phenotypes we selected 15 traits. We are performing SCZ-RPS analysis for each trait, so we employ a conservative alpha level (α=0.001). Based on the 99 low and 98 high SCZ-RPS groups we recruited from the larger distribution, we had 80% power to detect an association where R2 > 0.03 (Figure s1). In comparison, an opportunistic sample of SCZ-RPS would need to be exponentially larger (N = ~ 600; α = 0.001) to be adequately powered (>80%) to detect a similar effect.


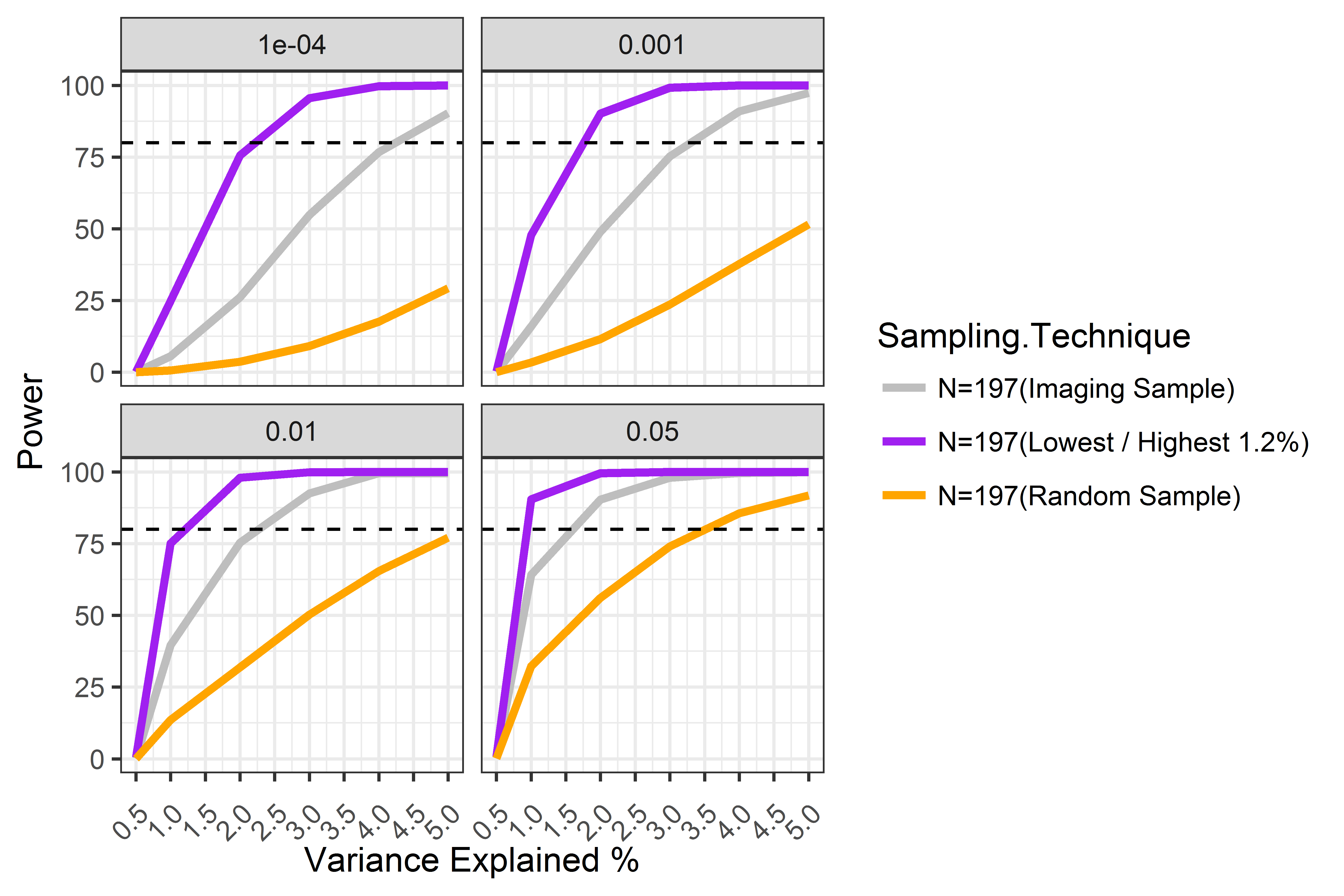


Figure s1. Power calculation for a) 197 individuals based on their actual SCZ-RPS rank (recall-by-genotype – in grey); b) a randomly drawn opportunistic sample 197 individuals (opportunistic sample – in orange) or c) the maximum power - if the highest and lowest 1.2% individuals (N=99 or 98 / 8365, respectively) ranked by SCZ-RPS were recruited (purple). Power is presented at varying effect sizes (explained variance (*R*^2^) ranging from 0.5 – 5%: x-axis) and across a range of alpha levels (represented in the 4 different plots).

**sM2. Reversal Learning Paradigm**


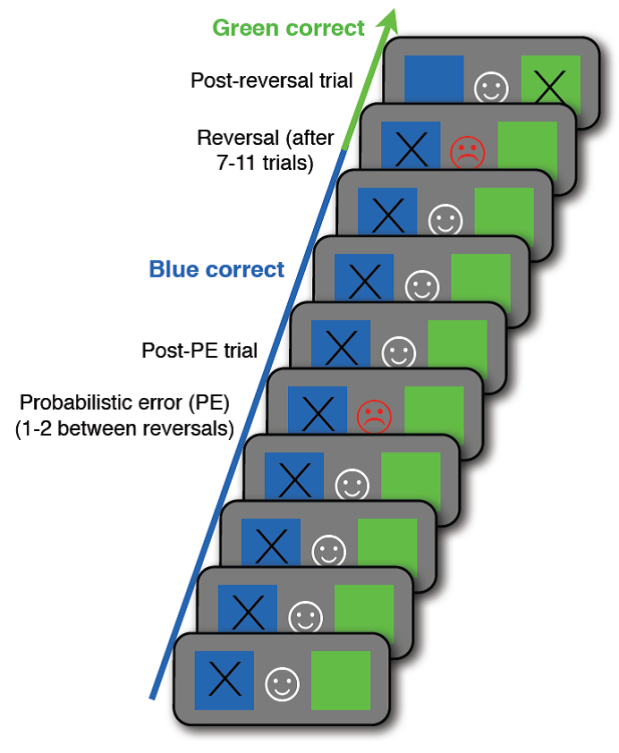


Figure s2. Probabilistic Reversal-learning Paradigm. For each trial, two stimuli were presented. Participants selected a green or blue square and feedback was presented as a positive or negative emoticon. BOLD was modelled in post-PE and post-reversal trials, which reflected choice behavior (shift > stay; after rule reversal) or choice outcome (reward > punishment) under high levels of uncertainty.

**Supplementary Results**

**sR1. Morphometric analysis**


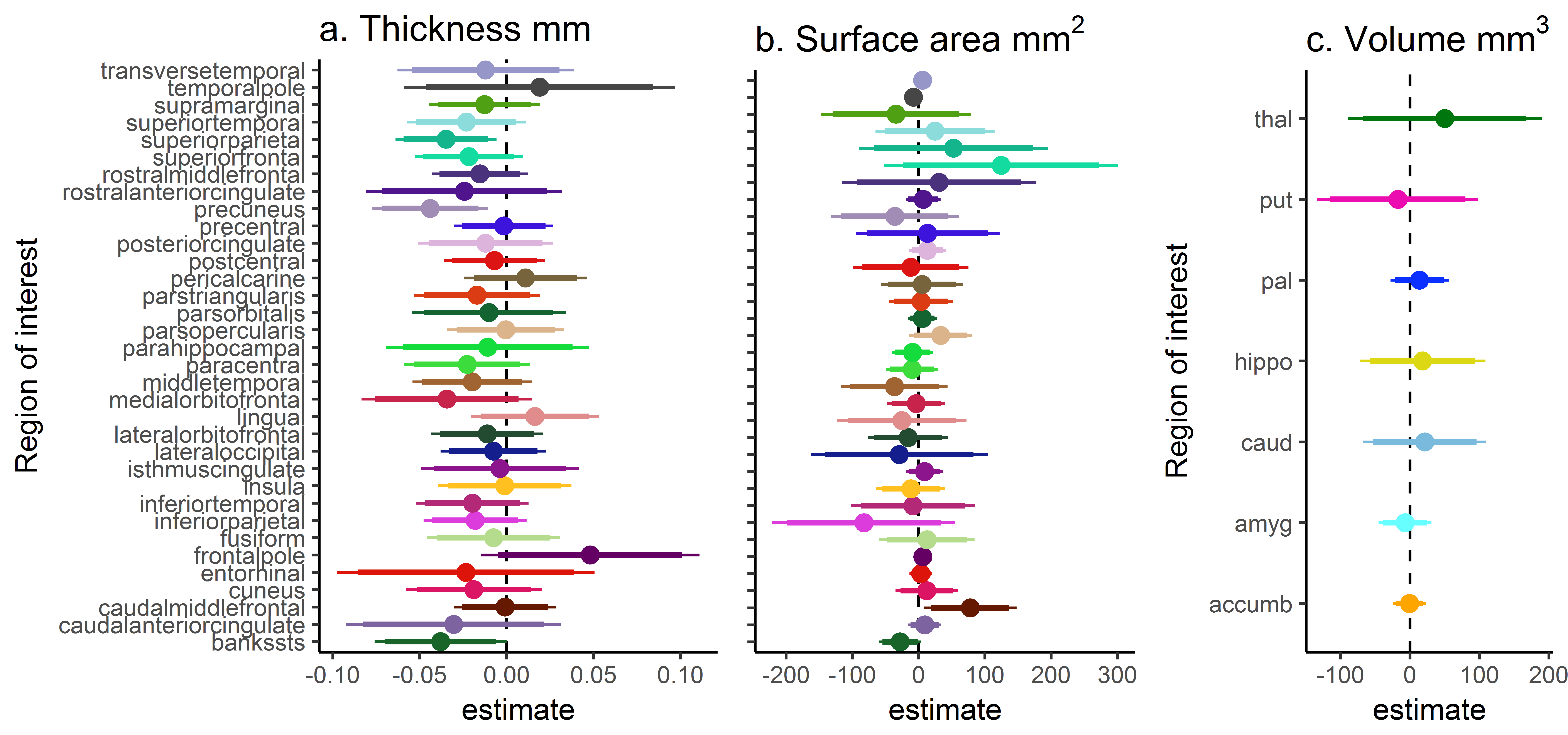


Figure s3. Coefficients (± 95% confidence intervals) for average cortical parcellation (for each of the 34 regions of interest and 7 subcortical volumes) by SCZ-RPS group (lower coefficients reflect an association between reduced a) thickness (mm), b) surface area (mm2) and c) subcortical volume (mm3) and high SCZ-RPS group allocation.

**sR2. Reversal Learning Performance**

SCZ-RPS groups (low vs high) were matched for performance at each trial type (Table S1). All results remained unchanged after inclusion of gender as a covariate.

| **Trial Type** | **SCZ-RPS (Low)** | **SCZ-RPS (HIGH)** | **t_(182)_** | **P** |
| --- | --- | --- | --- | --- |
| **Accuracy %)** |  |  |  |  |
| PE Trials | 82.91667 | 86.10372 | 1.3343 | 0.1838 |
| Post-PE (+1) | 47.22222 | 47.93883 | 0.20411 | 0.8385 |
| Post-PE (+2) | 71.81944 | 73.81205 | 0.75187 | 0.4531 |
| Reversal | 17.47473 | 16.82784 | -0.27966 | 0.7801 |
| Reversal (+1) | 61.31314 | 64.99034 | 1.1722 | 0.2427 |
| **Reaction Time (ms)** |  |  |  |  |
| PE Trials | 559.1528 | 559.6895 | 0.026309 | 0.9790 |
| Post-PE (+1/2) | 534.8514 | 546.9934 | 0.52757 | 0.5984 |
| Reversal | 533.2713 | 563.5035 | 1.4974 | 0.1360 |
| Reversal (+1) | 537.5380 | 566.1773 | 1.2671 | 0.2068 |

Table S1. Independent sample t-tests for low vs high SCZ-RPS during all trails type leading to and including switching reward contingencies.


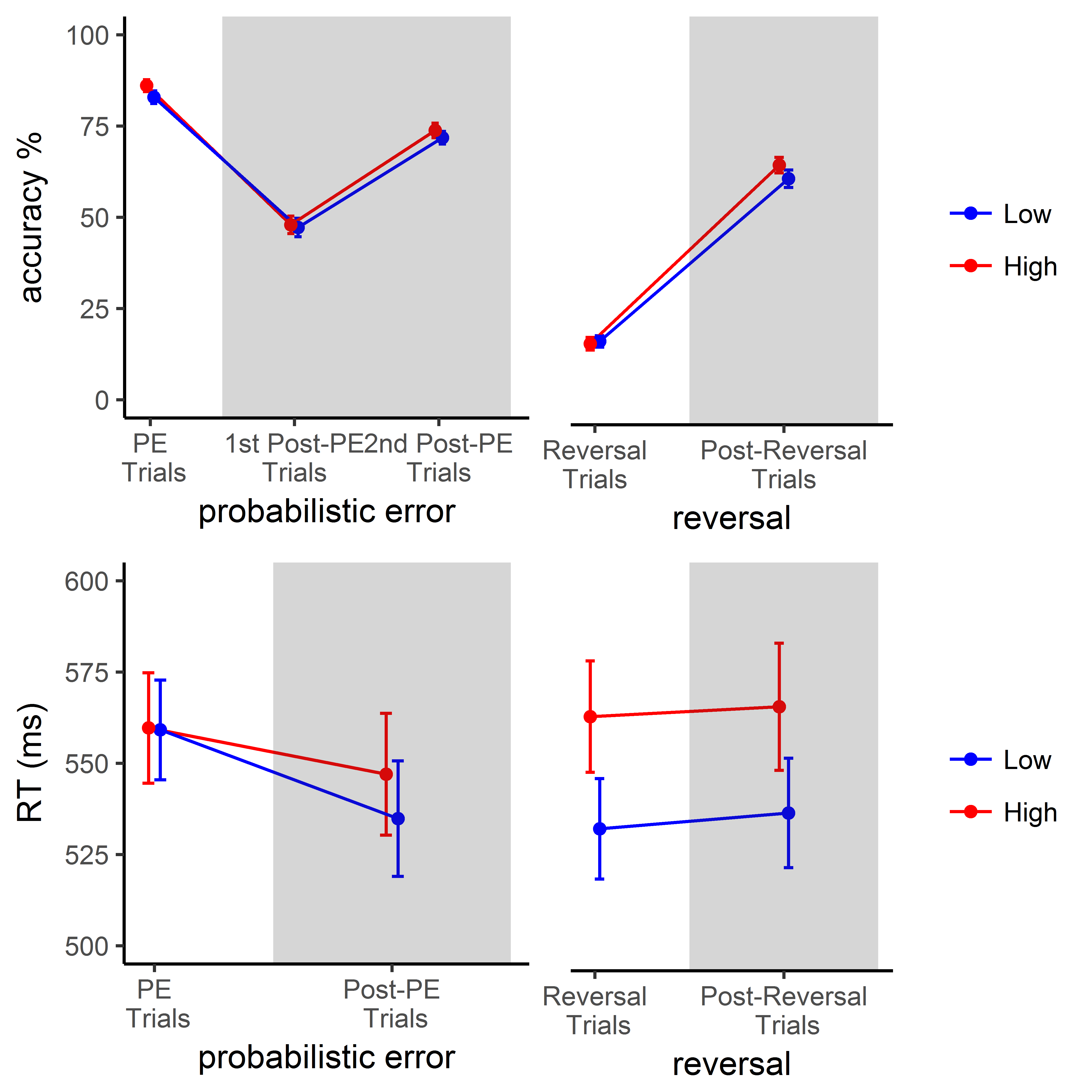


Figure s4. Low and high SCZ-RPS groups were matched for accuracy (%) and reaction time (RT) in ms (milliseconds) in events following a) a probabilistic error (PE) or b) following a reversal in reward contingency. Grey shading represents time points modelled in both the BOLD contrasts. Error bars reflect ±1 standard error of mean.
